# A Case of Sacral Ancient Schwannoma Presenting With Abdominal Pain

**DOI:** 10.7759/cureus.98406

**Published:** 2025-12-03

**Authors:** Muhammad Shariq Rahemtoola, Morgan Blake, Niall Pumfrey, Anas Abdallah Ibrahim Husein, Sohail N Malik

**Affiliations:** 1 Urology, North Manchester General Hospital, Manchester, GBR; 2 Internal Medicine, Manchester Foundation Trust, Manchester, GBR; 3 Urology, Manchester Royal Infirmary, Manchester, GBR; 4 General Surgery, North Manchester General Hospital, Manchester, GBR

**Keywords:** abdominal pain, prognosis, radiological surveillance, sacral ancient schwannoma, surgical resection

## Abstract

We report the case of a female in her sixties presenting to secondary care with sudden-onset abdominal pain. Comprehensive laboratory and imaging investigations were performed, exploring a broad range of differential diagnoses. These ultimately revealed a rare manifestation of an ancient schwannoma located in her sacrum. This case contributes to the limited literature on schwannomas presenting outside the cranium and upper limbs and highlights the diagnostic challenges associated with atypical tumor locations. It serves as an important opportunity to reflect on clinical practice, for example, the work-up for abdominal pain and the value of different imaging modalities in such cases. It also exemplifies the varying difficulty faced between centers in accessing specialist investigations and demonstrates the patient experience.

## Introduction

A schwannoma is a benign, slow-growing tumor arising from Schwann cells, which form the protective covering of peripheral nerves. A rarer subtype, accounting for approximately 1% of all schwannomas, is the ancient schwannoma, characterized by degenerative cytologic features and an even slower growth rate that often leads to delayed presentation [1].

Existing literature, which is limited on the topic, indicates that schwannomas can occur across the adult population without clear risk factors, although some studies suggest a slight predominance in females aged 40-60 years [2,3]. Most schwannomas typically present in the head and neck region (25%-50%), such as vestibular schwannomas affecting the eighth cranial nerve [4]. This is the most common form with an estimated incidence of 2.2 cases per 100,000 person-years in the United Kingdom [5]. They may also occur peripherally, including in the upper limbs (19%) and in the spine [3,6]. Rarely, schwannomas develop in other locations, including the retroperitoneum and sacral regions, which together account for less than 1% of cases (<0.5%-1%) for which there is limited epidemiological data [7,8].

Typical schwannomas are strongly associated with specific clinical symptoms, such as progressive unilateral hearing loss and tinnitus when in the eighth cranial nerve, and carpal tunnel syndrome when in the upper limbs. Symptoms in rarer locations vary on a case-by-case basis. In contrast, schwannomas arising in atypical locations may present variably or even remain asymptomatic. For example, sacral schwannomas have been reported as asymptomatic or as causing abdominal pain and polyuria, as well as early satiety and constipation [8,9]. Abdominal and lumbar pain of varying acuity has also been reported in lesions of the retroperitoneum and thoracic spine [3,4].

Given the insidious nature of ancient schwannomas, many are often found incidentally on imaging. To distinguish from other differentials, such as chordoma, neurofibroma, or gynecological malignancies, biopsy remains the diagnostic gold standard. Ancient schwannomas are identified histologically with nuclear atypia, cystic changes, hemorrhage, siderophages, as well as perivascular hyalinization, myxoid change, and calcification [2]. This can be confirmed immunochemically with S100 staining to confirm neural origins. Surgical excision is often the mainstay of treatment, although proximity to critical neural structures often necessitates careful consideration between operative management and active surveillance [8].

## Case presentation

A female in her sixties presented to the emergency department with sudden-onset, severe central abdominal pain radiating to the back and right iliac fossa. The pain had lasted approximately six hours and was described as constant and sharp. She reported feeling feverish and nauseous but denied any gastrointestinal or genitourinary symptoms or recent weight loss.

Her past medical history included hemiplegic migraines and hypertension, both well controlled with Topiramate (100 mg twice daily), Lisinopril (20 mg twice daily), and Sumatriptan (50 mg as needed). Her surgical history included a laparoscopic cholecystectomy in the 1990s and an emergency cesarean section in the 1980s. She lived with her partner, was a lifelong nonsmoker, consumed minimal alcohol, and was fully independent in activities of daily living (Eastern Cooperative Oncology Group (ECOG) performance status 0). There was no relevant family history.

On initial assessment, she was referred to the general surgery team for evaluation of an acute abdomen. Her vital signs were within normal limits except for a mild hypertension (150/101 mmHg). There were no peripheral stigmata of systemic disease on examination. Abdominal examination revealed generalized global tenderness without signs of peritonism, rebound tenderness, or guarding, and Rovsing, McBurney, or Psoas signs were all negative. Neurological examination of all four limbs was unremarkable, with no red-flag features suggestive of cord compression. Laboratory investigations and urinalysis remained within normal limits throughout her admission.

**Figure 1:**
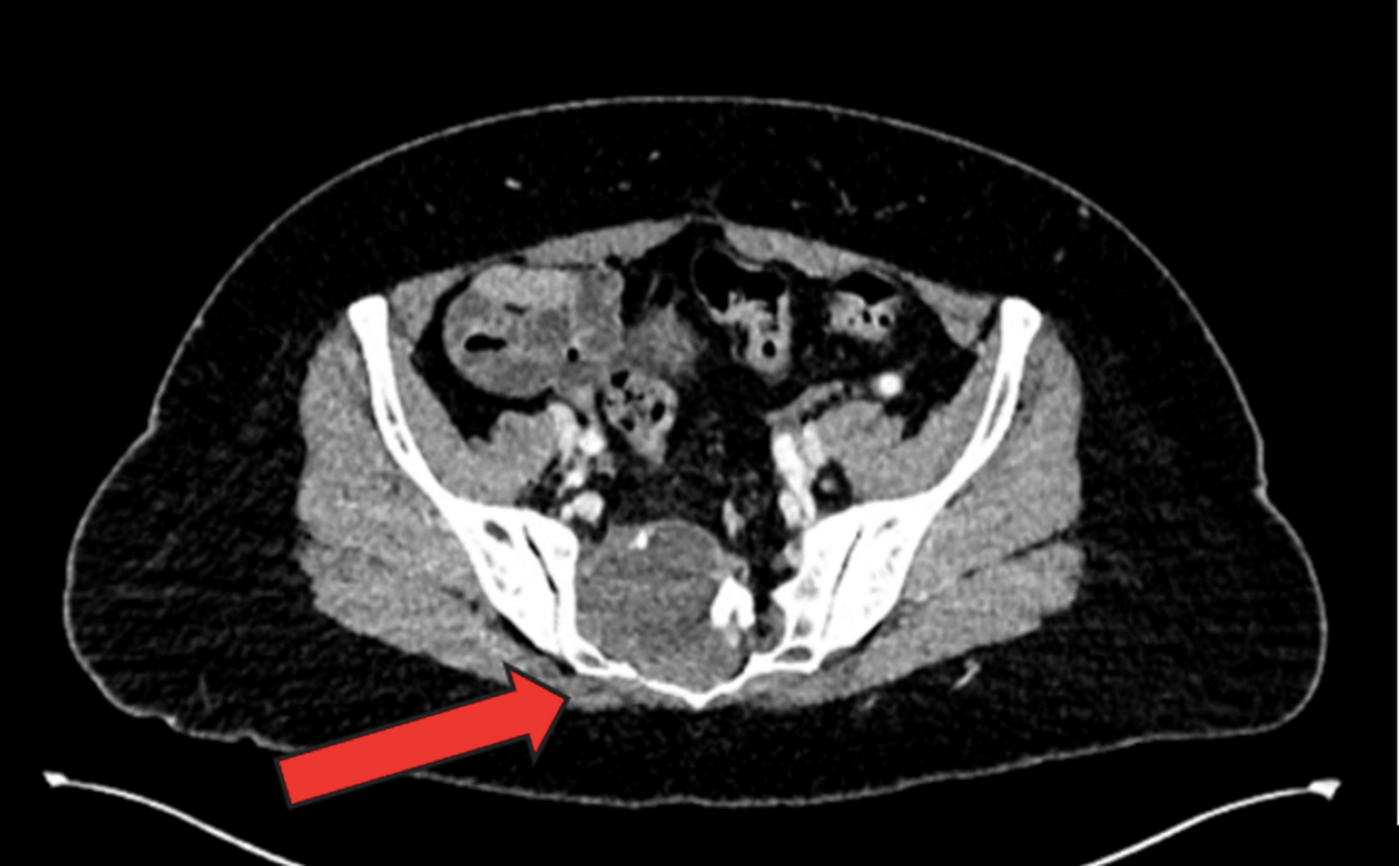
CT abdomen and pelvis (CT AP) demonstrating a right paracentral sacral mass with extra-osseous intra-abdominal extension, measuring approximately 5.5 × 3.6 cm, with mixed enhancing and non-enhancing areas and likely nerve root impingement (demarcated by red arrow).

To rule out initial differentials of appendicitis, abdominal aortic aneurysm (AAA), and ovarian pathology, a computed tomography (CT) scan of the abdomen and pelvis (Figure 1) was performed. This revealed a right paracentral sacral mass but was otherwise unremarkable, except for mild sigmoid diverticulosis.

**Figure 2:**
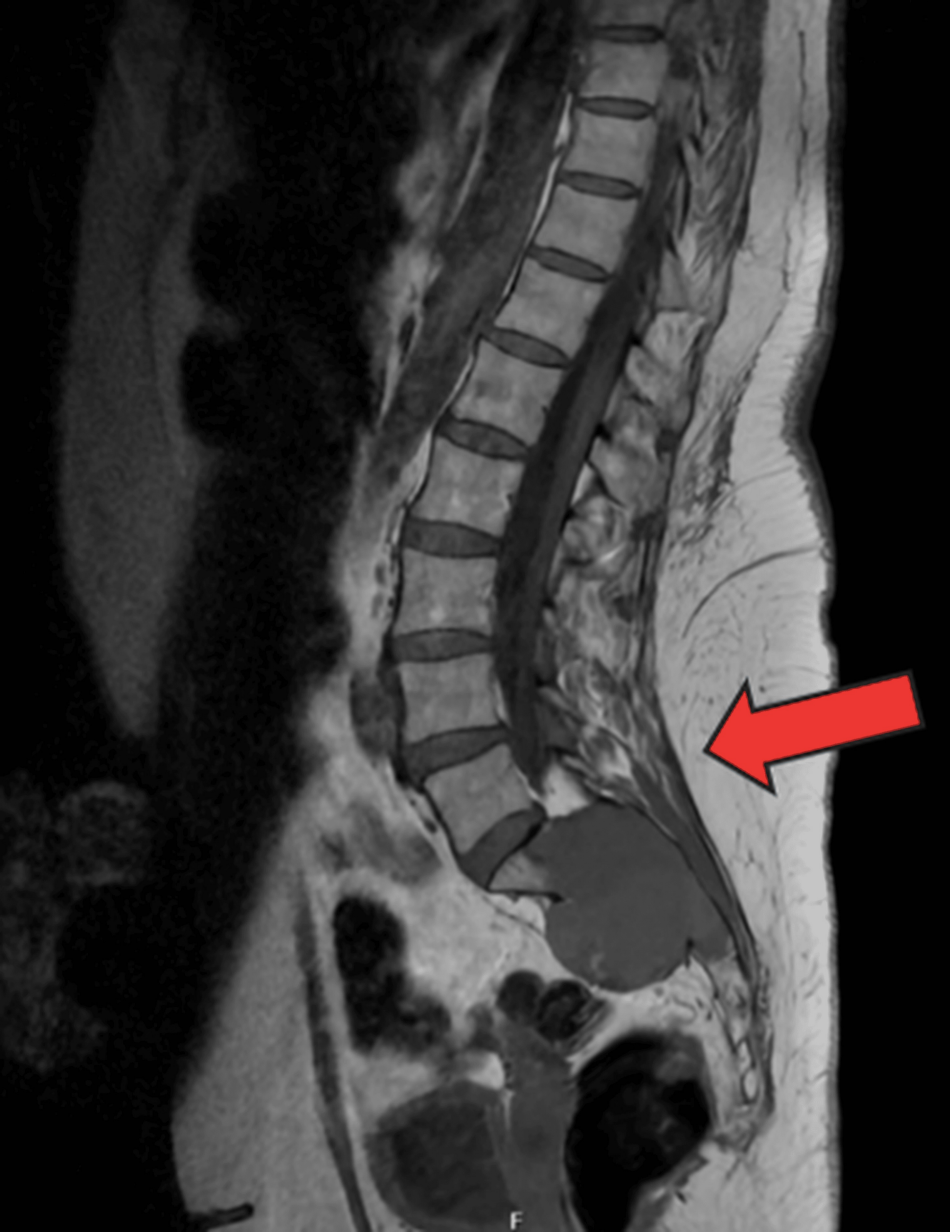
Magnetic resonance (MR) whole-spine sagittal view. MR whole spine with the red arrow demonstrating a multiseptated cystic sacral mass involving the right sacral ala, measuring 58 × 40 × 48 mm. The mass obliterates the sacral canal and the upper right sacral foramina, with compression of the sacral nerve roots at S1, S2, and S3. Additionally, the thecal sac is displaced to the left, and the tumor bulges into the anterior presacral space.

**Figure 3:**
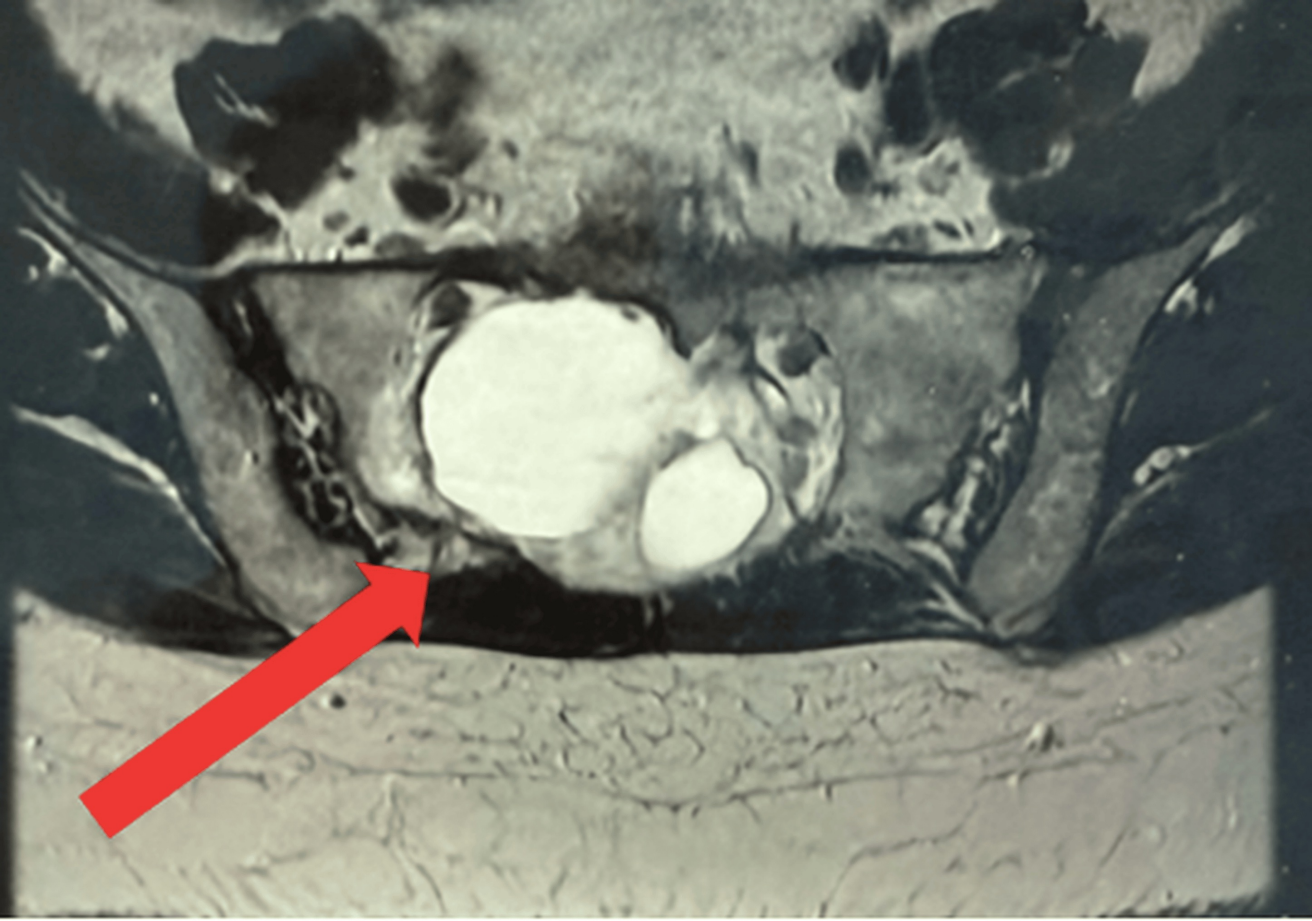
Magnetic resonance (MR) whole spine (axial view). Red arrow pointing to a multiseptated cystic sacral mass involving the right sacral ala, measuring 58 × 40 × 48 mm.

**Figure 4:**
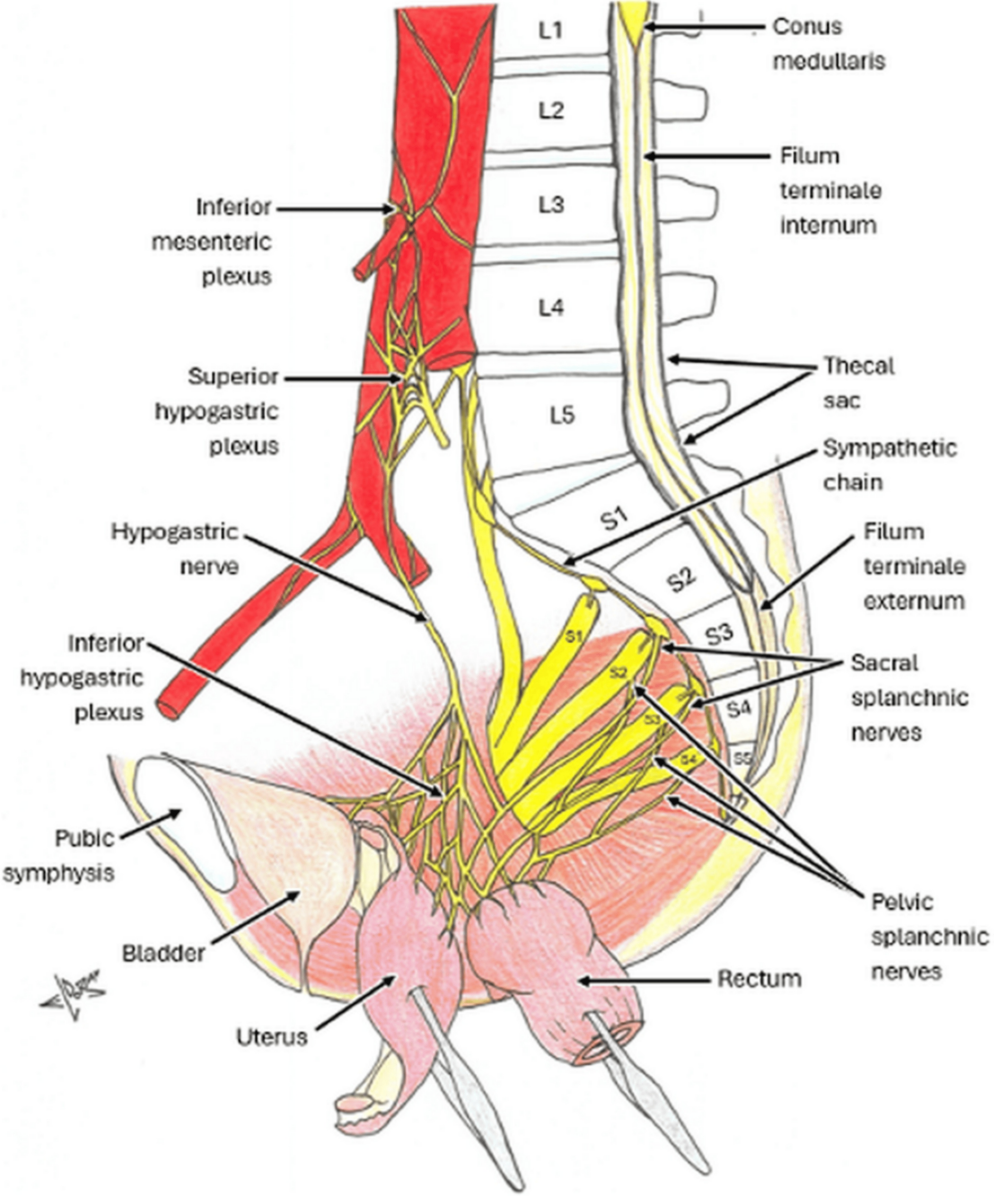
Illustrative diagram demonstrating pelvic anatomy and nervous supply for reference. Image credit: Morgan Blake.

Subsequently, an urgent magnetic resonance imaging (MRI) scan of the whole spine was obtained (Figures 2, 3) on the advice of the regional neurosurgery team. It demonstrated a multiseptated cystic sacral mass, with differential diagnoses including chordoma, giant cell tumor, and metastasis. For reference, the normal local anatomy is illustrated in Figure 4.

**Figure 5:**
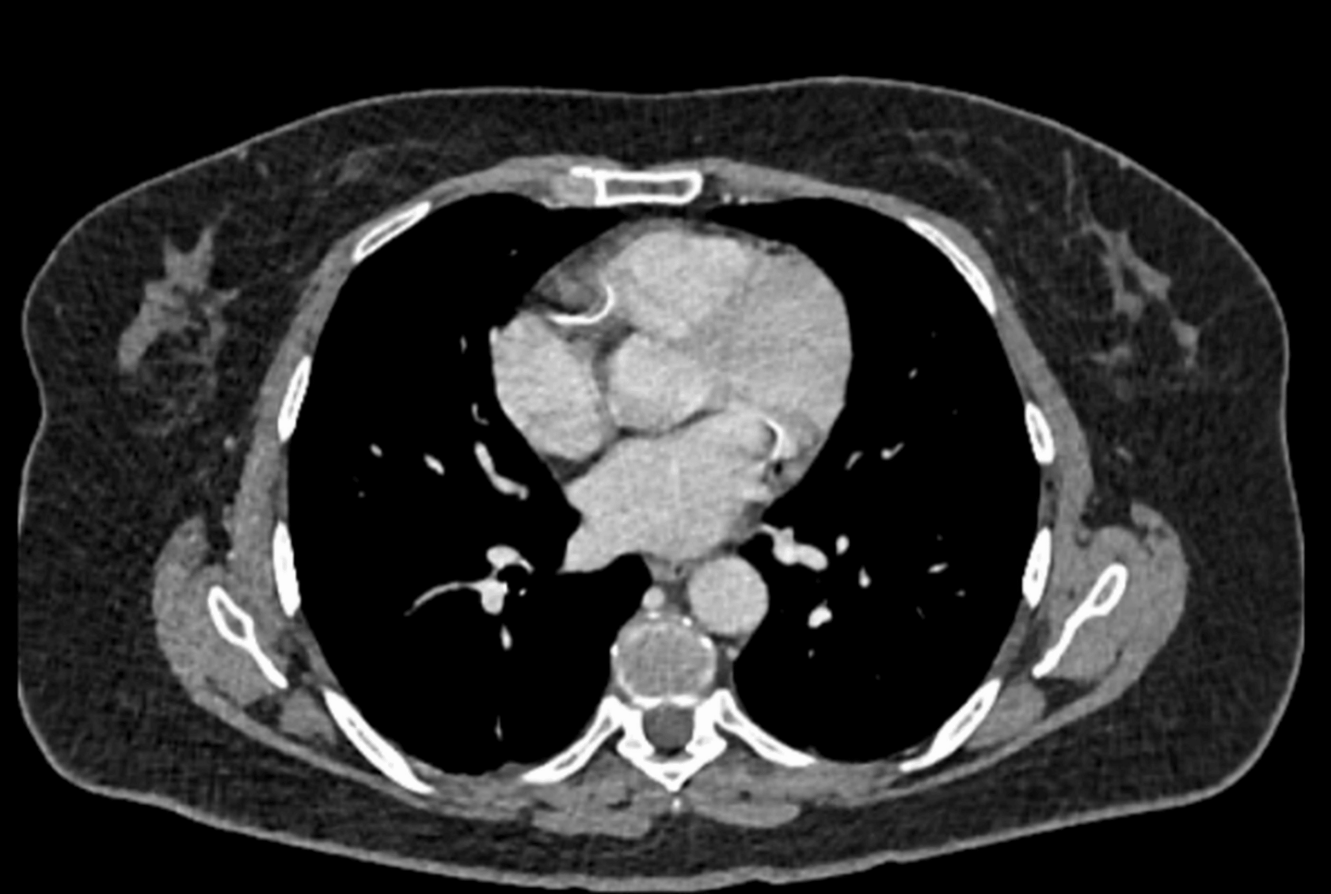
Axial computed tomography (CT) scan of the thorax demonstrating no evidence of metastatic disease.

A CT scan of the thorax was then performed to exclude metastatic disease, and this was unremarkable (Figure 5). The patient was referred urgently to the United Kingdom’s Tertiary Specialist Unit. Upon review at the specialist center, her abdominal pain had resolved. The likely diagnosis was explained to the patient, and no other red flag features were identified in the history or examination at that time.

**Figure 6:**
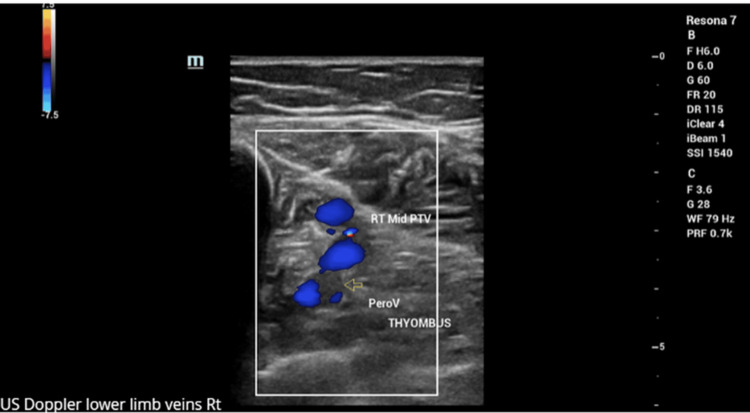
Ultrasound Doppler confirming the presence of deep-vein thrombosis.

To confirm the suspected diagnosis, a CT-guided biopsy of the sacral lesion was carried out. While awaiting histology results, the patient presented to Ambulatory Care with symptoms consistent with deep-vein thrombosis (DVT). This was confirmed on imaging (Figure 6) and was managed successfully with apixaban 5 mg twice daily for one week.

The biopsy sample, consisting of a 15-mm tan and pale core alongside multiple tan and hemorrhagic tissue fragments, confirmed the suspected diagnosis. Microscopy showed a moderately cellular lesion composed of spindle cells with monomorphic nuclei and indistinct cytoplasmic borders, without mitoses. Immunostaining demonstrated strong positivity for SOX10 and S100, preserved expression of H3K27me3, and negative staining for CD34, smooth muscle actin (SMA), and desmin.

Given the slow-growing nature of the tumor and its proximity to neural structures, and as the patient is otherwise stable without significant disease burden, the tumor will be monitored with active surveillance through MR imaging and is not planned for excision.

## Discussion

This case presents an example of a sacral ancient schwannoma manifesting as abdominal pain in a female in her 60s. This is a rare pathology with few documented cases, and as such, this case adds to a limited pool of literature. It also provides an opportunity to explore overlap with other cases of ancient schwannoma and to reflect on key lessons for clinical practice.

Ancient schwannoma in itself is a rare form of schwannoma with specific histological features. In conjunction with the sacrum being an extremely infrequent location for manifestation means this is a rare case with very few similar examples. This ancient schwannoma presented as a cystic sacral mass with involvement of the right sacral ala, obliterating the sacral canal and upper right sacral foramina, with compression of the sacral nerve roots at the right S1, S2, and S3 nerve roots. The rarity of the sacrum as a point of growth is likely due to the pathophysiology of ancient schwannomas occurring in peripheral nerves, which most commonly appear in the head, neck, and limbs. Sacral involvement may be less common, as there are fewer large peripheral nerves here, with a narrow sacral spinal canal with relatively fewer nerve roots. The slow-growing, insidious, and often asymptotic nature of these tumors, combined with the imaging and histological diagnostic challenges, may also contribute to the low incidence of reporting. 

Similar to another reported sacral manifestation, this patient also presented with abdominal pain, though without the polyuria and incomplete emptying, or indeed the satiety and distention of another case [8,9]. Interestingly, abdominal pain of a chronic and acute nature, respectively, was also reported in the cases found in the retroperitoneum and thoracic spine [4,10]. It is therefore evident that though symptoms are generally related to tumor location, these vary between cases, and there is no definitive presentation of ancient schwannomas. Abdominal pain in this case was likely caused by a combination of mass effect, nerve impingement, and referred pain to the viscera. The Schwannoma compressed the S2-S4 nerve roots, which ultimately innervate aspects of the lower abdominal organs. Nerve compression in this region typically causes deep pelvic pain, which can be interpreted as abdominal in nature by the patient, as also seen in a case involving the femoral nerve [7]. This pain was likely worsened in this case through mass effect by the tumor extending anteriorly into the presacral space and displacing the thecal sac. 

As with other examples, CT and MR imaging were the mainstay for investigative imaging in this case. CT imaging in isolation may not be sufficient to identify a precise tumor type; for example, the radiological report listed chordoma as the primary differential, alongside metastases from an unknown primary. Furthermore, it may not fully exclude the presence or extent of nerve involvement. MR imaging in this case, although not available for a few days, ultimately indicated an Ancient Schwannoma through the characteristic tumor features. It was also able to clarify the extent of the nerve involvement, which guides the acuity of further intervention. Ancient schwannomas are ultimately diagnosed at a gold standard through histological analysis, which shows hyalinization, nuclear atypia, and hemosiderin deposition without conspicuous mitotic activity [2]. In this case, biopsy demonstrated monomorphic nuclei, indistinct cytoplasmic borders without necrosis, and strong immunostaining positivity for SOX10 and S100 markers, confirming the diagnosis. 

For most cases, surgical intervention is considered the mainstay of treatment, though it is ultimately guided by symptoms and tumor characteristics. For example, consideration should be given to proximity to organs and their nervous supply. In one prospective study, patients with Schwannomas were treated based on the extent of symptoms, with asymptomatic patients monitored over time with radiological surveillance [11]. In the literature, surgical resection was carried out without complication with an excellent prognosis. In this case, given proximity to nervous structures and the lack of disease burden, the patient will be managed through observation with further MR imaging and not surgical intervention. While ultimately reassuring, the psychological impact of living with a slow-growing tumor should not be underestimated; the patient has been offered ongoing support.

This case underscores the broad differential diagnosis for non-specific abdominal pain and the importance of imaging, which will ultimately rule out rare and atypical pain. CT should always be considered in patients presenting with atypical abdominal pain without previous imaging, particularly in the context of their well-tolerated, low-risk profile [12]. Clinicians must interpret imaging findings in conjunction with the patient’s history and examination, including neurological assessment, to ensure rare but serious pathologies are not overlooked. Rare presentations such as sacral schwannoma should be managed through a multidisciplinary team (MDT) approach and, where necessary, escalated via national referral pathways. 

## Conclusions

This case highlights a rare sacral manifestation of an ancient schwannoma, a tumor subtype with an already remarkably low incidence. The patient presented with symptoms of an acute abdomen, underscoring the broad differential diagnosis for abdominal pain and the importance of thorough imaging when clinical findings are inconclusive. Imaging and histological features were consistent with previously reported cases, though management in this instance favored active surveillance due to symptom resolution and the lesion’s proximity to neural structures. This case reinforces the value of biopsy as the diagnostic gold standard and the need to balance surgical risk against potential benefit.
