# Correction: A Case of Sacral Ancient Schwannoma Presenting With Abdominal Pain

**DOI:** 10.7759/cureus.c417

**Published:** 2026-03-26

**Authors:** Muhammad Shariq Rahemtoola, Morgan Blake, Niall Pumfrey, Anas Abdallah Ibrahim Husein, Sohail N Malik

**Affiliations:** 1 Urology, North Manchester General Hospital, Manchester, GBR; 2 Internal Medicine, Manchester Foundation Trust, Manchester, GBR; 3 Urology, Manchester Royal Infirmary, Manchester, GBR; 4 General Surgery, North Manchester General Hospital, Manchester, GBR

The following acknowledgement has been added to this article: "The authors would like to thank Eldora Sequeira for her time in designing Figure 4."

